# Fault Diagnosis of Rolling Bearings Based on a Residual Dilated Pyramid Network and Full Convolutional Denoising Autoencoder

**DOI:** 10.3390/s20205734

**Published:** 2020-10-09

**Authors:** Hongmei Shi, Jingcheng Chen, Jin Si, Changchang Zheng

**Affiliations:** Key Laboratory of Vehicle Advanced Manufacturing, Measuring and Control Technology, Beijing Jiaotong University, Beijing 100044, China; 19125959@bjtu.edu.cn (J.C.); 18116040@bjtu.edu.cn (J.S.); 19121290@bjtu.edu.cn (C.Z.)

**Keywords:** intelligent fault diagnosis, residual learning, dilated pyramid network, fully convolutional denoising autoencoder, noise robustness

## Abstract

Intelligent fault diagnosis algorithm for rolling bearings has received increasing attention. However, in actual industrial environments, most rolling bearings work under severe working conditions of variable speed and strong noise, which makes the performance of many intelligent fault diagnosis methods deteriorate sharply. In this regard, this paper proposes a new intelligent diagnosis algorithm for rolling bearing faults based on a residual dilated pyramid network and full convolutional denoising autoencoder (RDPN-FCDAE). First, a continuous wavelet transform (CWT) is used to convert original vibration signals into time-frequency images. Secondly, a deep two-stage RDPN-FCDAE model is constructed, which is divided into three parts: encoding network, decoding network and classification network. In order to obtain efficient expression of data denoising feature of encoding network, time-frequency images are first input into the encoding-decoding network for unsupervised pre-training. Then pre-trained coding network and classification network are combined into residual dilated pyramid full convolutional network (RDPFCN) for parameter fine-tuning and testing. The proposed method is applied to bearing vibration datasets of test rig with different speeds and noise modes. Compared with representative machine learning and deep learning method, the results show that the algorithm proposed is superior to other methods in diagnostic accuracy, noise robustness and feature segmentation ability.

## 1. Introduction

In the big data-driven industrial era, fault diagnosis technology has become the core part of prognostics and health management (PHM), which plays an significant role in the maintenance of intelligent mechanical devices [1]. With the constant development of artificial intelligence technology, research on rotating machinery fault diagnosis has taken new directions and produced notable breakthroughs [2]. Rolling bearings, as the key components of rotating machinery and equipment, are prone to failure, and research on bearings fault diagnosis has an important guiding significance for the reliability, safety and economy of the entire machinery and equipment [3,4]. Under practical operating conditions, however, rolling bearings often operate in harsh environments involving variable speeds and high noise. Faced with such a complex and changing industrial environment, it is very challenging to carry out fault diagnosis research.

Traditional fault diagnosis methods based on machine learning have attracted extensive attention in the past two or three decades [5]. It can be summarized into three steps: sensor signal acquisition, feature extraction and selection and fault classification. Feature extraction and selection is a labor-intensive process, which often requires a lot of signal processing technology and artificial experience to judge, including time domain feature analysis, frequency domain feature analysis, cepstrum analysis, wavelet transform (WT) and empirical mode decomposition (EMD), etc. Then these statistical features are input into a machine learning classifier for fault diagnosis, such as K-nearest neighbor (KNN), support vector machine (SVM), random forest (RF), gradient boosting decision tree (GBDT) and artificial neural network (ANN), etc. [6,7,8,9,10]. Bhakta et al. used cepstrum analysis to pre-process the rolling bearing datasets provided by the Case Western Reserve University (CWRU) laboratory, then used the gradient boosting (GB) learning algorithm for fault diagnosis, and achieved good results [11]. Qin et al. decomposed the original signal of rolling bearings into several inherent mode functions (IMF) through ensemble empirical mode decomposition (EEMD), and then selected an effective IMF and input its energy entropy as a feature to the random forest classifier for fault diagnosis [12]. Based on the CWRU bearing datasets, Moosavian et al. employed discrete wavelet transform (DWT) to extract signal features and compared the diagnostic performance of ANN, SVM and least square SVM (LS-SVM) classifiers, and concluded that LS-SVM has the best Diagnostic effect. However, these intelligent fault diagnosis methods still have great limitations [13]:

- The diagnostic accuracy of these methods to a large extent depends on feature extraction engineering and relies heavily on the a priori knowledge and expert knowledge of signal processing techniques. If these manually extracted features are insufficient to complete the task, the accuracy of fault diagnosis will be significantly reduced. The manual extraction process is also very time-consuming and laborious.
- The feature extraction and selection are different for each specific fault diagnosis task, which indicates that it is difficult to design a set of feature extractors with strong generalization ability.
- Traditional machine learning algorithm has a simple structure and a shallow layer, which limits the classifier’s ability to learn more complex nonlinear relationships in fault diagnosis tasks, and it cannot extract deep-level fault feature information.

Deep learning (DL) algorithm provides an effective solution to the above problems because of its powerful ability to automatically learn complex functions. Deep learning technology can continuously improve its performance with the increase of data scale, while in traditional machine learning algorithms it is difficult to use massive data to continuously improve their performance. DL has shown great application prospects in many research fields [14,15,16]. For example, DL’s performance in image classification, object detection, semantic segmentation and other tasks in the field of computer vision greatly surpasses traditional methods, while in the field of natural language processing has played an irreplaceable role in the research of tasks such as speech recognition, machine translation, and dialogue systems, and has also achieved breakthrough results in autonomous driving, medical health, and fault diagnosis.

In recent years, deep learning methods have been successfully applied to the health monitoring of mechanical equipment [17,18,19,20,21]. Hao et al. Proposed an one-dimensional convolutional long short-term memory (LSTM) networks, where both the spatial and temporal features of multisensor measured vibration signals are extracted and then jointed for better bearing fault diagnosis [22]. Xue et al. proposed a fault diagnosis method based on a deep convolution neural network (DCNN) and SVM, it achieved good accuracy [23]. Xu et al. developed a stacked denoising autoencoder (SDAE) to extract the feature of the bearing diagnostic signal, and then used the Gath-Geva (GG) clustering algorithm for fault diagnosis [24]. Zhu et al. first proposed the cyclic spectral coherence analysis (CSCoh) method to obtain a CSCoh diagram of the rolling bearing vibration signal, and established a CNN model to learn the features for classification [25]. Xu et al. proposed a bearing fault diagnosis method based on CNN and RF ensemble learning, using multiple hierarchical features extracted by CNN model training, and performing diagnosis through the integration of multiple RF classifiers, achieving high diagnostic accuracy [26]. Zhao et al. proposed a Local Feature-Based Gated Recurrent Unit Networks (LFGRU) method to estimate the remaining life of the machine and perform fault diagnosis on the gearbox and rolling bearings [27]. Shao et al. designed a series of autoencoders with different activation functions to construct ensemble deep autoencoders (EDAEs) to learn the characteristics of vibration signals and use a combined strategy for bearing fault diagnosis [28]. Kong et al. also designed several DAEs with different activation functions and performed fault diagnosis of rolling bearings through the integration of models [29].

However, for the fault diagnosis of rolling bearings, the above methods still have certain deficiencies, the robustness of the model to noise has not been verified or the verification effect is not particularly ideal. In an industrial environment, rolling bearings operate in a background containing a lot of noise. These noises tend to cover part of the fault information, especially strong noise, which severely degrades the diagnostic performance of the various models [30,31]. Therefore, in order to improve the generalization ability of the model, the diagnostic algorithm should have strong anti-noise ability. Liang et al. designed a novel and high-accuracy fault detection approach named WT-GAN-CNN for rotating machinery, This model was based on CWT, generative adversarial nets (GANs) and CNN, the built CNN model is used to accomplish the fault detection of rotating machinery by the original training time-frequency images and the generated fake training time-frequency images, and verified its anti-noise ability through experiments [32]. Wang et al. combined GANs with stacked denoising autoencoder (SDAE), using BP neural network (BPNN) as the generator to generate samples, and SDAE was used as the GAN discriminator to diagnose the planetary gearbox, The model has a certain anti-noise performance [33]. Peng et al. proposed a new deep one-dimensional convolutional neural network (Der-1DCNN) based on one-dimensional residual blocks. The network has very good diagnostic performance and generalization ability [34]. Zhang et al. proposed using deep convolutional neural networks with wide first-layer kernels (WDCNN) method for bearing fault diagnosis, which mainly used the high frequency noise suppression mechanism of wide first-layer kernels [35]. In order to strengthen the anti-noise ability of the network, Zhang et al. also set the first layer of convolution kernel to a wide convolution kernel, and used the dropout method to construct a convolution neural networks with training interference (TICNN) model for rolling bearing fault diagnosis [36]. They also proposed a bearing fault diagnosis method based on a fully-connected winner-take-all autoencoder, which limits the maximum activation rate of each neuron of the sample, and uses soft voting to classify the collection, The model shows has a certain noise robustness [37]. Yao et al. proposed an intelligent bearing fault diagnosis method based on Stacked Inverted Residual Convolution Neural Network (SIRCNN), which through the application of depthwise separable convolution and inverted residual structure to ensure the lightweight of the model and accuracy in noisy environments [38].

At present, the deconvolution method based on convolution has not been widely used in the fault diagnosis of rolling bearings in environments with strong noise. Most denoising operations are based on a fully connected method. The convolution operation can retain the neighborhood and local features of space, and it can achieve weight sharing, thereby greatly reducing the number of parameters, and it has achieved leaps and bounds in the field of image processing.

In order to improve the processing ability of model information and the ability to extract depth denoising features, this paper proposes a new intelligent fault diagnosis method for rolling bearings, which is based on residual dilated pyramid network and full convolutional denoising autoencoder (RDPN-FCDAE). This method is dedicated to enhance the ability of model feature extraction and reduce the impact of environmental noise and complex working conditions, so as to achieve better fault diagnosis performance. The main contributions of this paper are summarized as follows:

- A features pyramid network structure with dilated convolution of different convolution kernel ratios and dilated rates is designed, which fully integrates the details and lager scale feature map information extracted by convolution kernels with different dilated coefficients, which is conducive to the network’s grasp of the overall features. Compared with the wide convolution kernel, the proposed network increases the receptive field of the convolution kernel without increasing the network parameters.
- Residual learning is used to effectively solve the problems of gradient vanishing, gradient exploding and performance degradation caused by the increase of network depth. In addition, RDPN-FCDAE has no max-pooling layer, which is replaced by stride convolutional layer to reduce the loss of weak fault information.
- A two-stage fault diagnosis algorithm based on fully convolutional denoising autoencoder is designed, which is divided into two stages of pre-training and fine-tuning. The pre-training of the autoencoder effectively improves the model’s ability to express denoising features, and it is helpful for the network to extract the weak fault features covered by noise. The fine-tuning uses a global average pool instead of a fully connected layer, which reduces the model parameters and can achieve the effect of any input size.

The rest of the paper is organized as follows: The second section introduces the theoretical background of the method. RDPN-FCDAE model and general steps of using the model for fault diagnosis are described in detail in the third section. Validation of the model through experiments is presented in Section 4. Conclusions are summarized in Section 5.

## 2. Methodologies

### 2.1. Time-Frequency Imaging

Time-frequency images can reflect the characteristics of the signal both in both the time domain and the frequency domain. The time-frequency imaging method is used to convert the original time series signals collected by sensors into the time-frequency domain for analysis. This is a kind of fault diagnosis for rotating machinery. The commonly used time-frequency imaging methods include short-time Fourier transform (STFT), CWT, Hilbert-Huang Transform (HHT) and so on [20]. Among them, CWT is widely used for feature extraction in fault diagnosis tasks, and can be used as a mathematical tool to convert the time series of signals to other feature spaces. In this paper, CWT is used to convert the original vibration signal of the bearing dataset into a time-frequency image expressed in grayscale.

The wavelet transform replaces the infinitely long trigonometric function base with a finite-length decaying wavelet base, which has scale variables and time variables, so it is very effective for non-stationary signals and transient signals.

Wavelet basis function ψ(t) is usually called the mother wavelet function. On this basis, a class of wavelet families can be obtained by scaling and translation, as shown below:

(1) $$\psi_{a,b}\left(t\right)={\left|a\right|}^{\frac{1}{2}}\psi \left(\frac{t-b}{a}\right)\;a,b\in R,a>0,$$

where *a* and *b* represent scale parameters and translation parameters, respectively, the scale parameter *a* changes the oscillation frequency by stretching or compressing the wavelet function, and the translation parameter *b* changes the position of the time window.

For any signal function f(t), the mathematical definition of CWT is as follows:

(2) $$W_{f}\left(a,b\right)=\langle f\left(t\right),\psi_{a,b}\left(t\right)\rangle ={\left|a\right|}^{-\frac{1}{2}}\int_{-\infty }^{+\infty }f\left(t\right)\psi^{*}\left(\frac{t-b}{a}\right)dt,$$

where $\psi^{*}\left(\cdot \right)$ is the complex conjugate function of the mother wavelet function ψ(⋅), and $W_{f}\left(a,b\right)$ is the inner product of the signal functions f(t). and $\psi_{a,b}\left(t\right)$. Based on the above operation, the signal f(t) is transformed by the wavelet family and projected into two dimensions of time and scale, so that the one-dimensional time series is converted into a two-dimensional time-frequency image.

### 2.2. Dilated Convolution

Dilated convolution is an optimization of ordinary convolution. A large-scale sparse matrix is used to replace the traditional convolution kernel. Yu and Koltun first proposed the concept of dilated convolution [39]. Compared with ordinary convolution, dilated convolution has a larger receptive field while keeping the kernel parameters unchanged, so the feature map information extracted through the convolution kernel also increases accordingly. The dilated convolution has a dilated rate, which is used to express the scale of the convolution kernel dilated. The mathematical definition of the two-dimensional dilated convolution can be expressed as Equation (3):

(3) $$y\left(m,n\right)=\sum_{i=1}^{M}\sum_{j=1}^{N}x\left(m+r\times i,n+r\times j\right)w\left(i,j\right),\;$$

where y(m,n) is the output of the dilated convolution, and *M* and *N* are the length and width of the convolution kernel. w(i,j) is the weight, and r is the dilated rate.

Assuming that the size of the original convolution kernel is *k × k*, the size of the convolution kernel with dilated rate r can be expressed as Equation (4):

(4) $$k^{\prime }=k+\left(k-1\right)\left(r-1\right),$$

**Figure 1:**
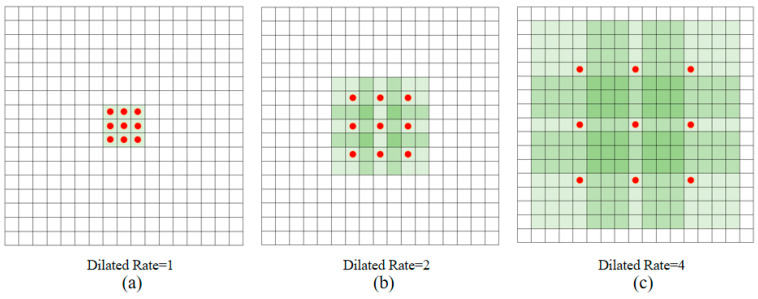
3 × 3 convolution kernel with different dilated rate.

For a given two-dimensional image, when the dilated rate *r* = 1, the dilated convolution is the same as the ordinary convolution. When the dilated rate *r* = 2, it means that each input skips one pixel. When the dilated rate *r* = 3, it means that 2 pixels are skipped. Figure 1 shows the dilated convolution on the two-dimensional data. The red dots represent the 3 × 3 convolution kernel input, and the green area is the receptive field captured by these inputs. Figure 1a represents the feature map obtained by the original image through the dilated convolution of *r* = 1 (ordinary convolution), and its receptive field is 3 × 3; Figure 1b represents the feature map obtained by Figure 1a through dilated convolution of *r* = 2, and its receptive field is 7 × 7; Figure 1c represents the feature map obtained by Figure 1b through dilated convolution of *r* = 4, and its receptive field is 15 × 15.

### 2.3. Residual Learning

**Figure 2:**
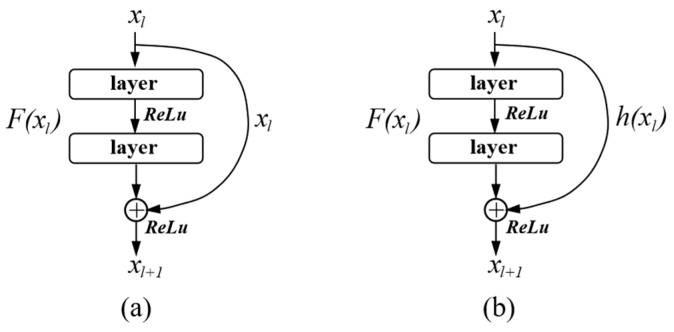
Architecture of residual block. (**a**) Identity-block. (**b**) Conv-block.

As the number of network layers increases, a network composed of multiple non-linearly activated convolutional layers can fit a very complex function, so the stronger the ability of the model to express features. However, the deep network has difficulties in training and performance degradation. He et al. First proposed the concept of a residual network [40], and proved that it can adilated the problem of gradient vanishing and gradient exploding in the back propagation process. The residual connection makes the deep network easier to optimize. Residual network is composed of a series of residual blocks, which introduces a shortcut connection. Figure 2 shows the residual block structure. 

In Figure 2a, the residual block is divided into an identity mapping part and a residual part, $x_{l}$ is a direct mapping, $F\left(x_{l},W_{l}\right)$ is the residual part, which is generally composed of two or more convolution operations. At this time, the output of the residual block can be expressed as Equation (5):

(5) $$x_{l+1}=f\left(x_{l}+F\left(x_{l},W_{l}\right)\right),$$

where *f* represents the activation function, and the linear rectifier unit (ReLU) function is generally used.

However, the dimensions of the feature maps of $x_{l}$ and $x_{l+1}$ may be different. At this time, 1 × 1 convolution needs to be used for dimension matching, as shown in Figure 2b, where $h\left(x_{l}\right)=W_{l}^{\prime }x$, and the output of residual block is described as:

(6) $$x_{l+1}=f\left(h\left(x_{l}\right)+F\left(x_{l},W_{l}\right)\right),$$

Residual network through the shortcut connection operation makes the cross-layer flow of information possible, the network is easy to optimize, and the training speed is significantly improved.

### 2.4. Convolutional Denoising Autoencoder (CDAE)

**Figure 3:**
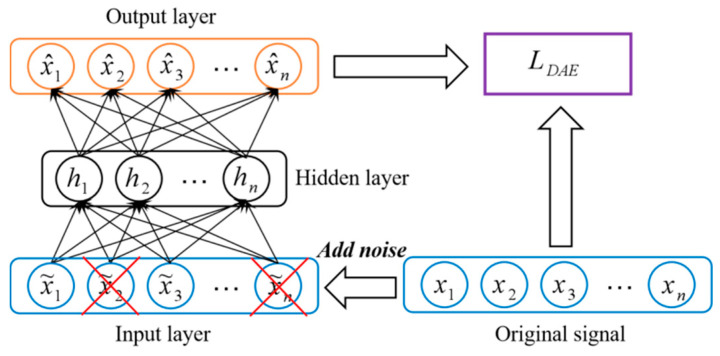
The structures of standard DAE.

The denoising autoencoder (DAE) integrates the denoising code into the traditional autoencoder [41]. It accepts damaged input data, and reconstructs the original uncorrupted data as output through training to enhance the network’s anti-noise ability, the structure of DAE is shown in Figure 3.

**Figure 4:**
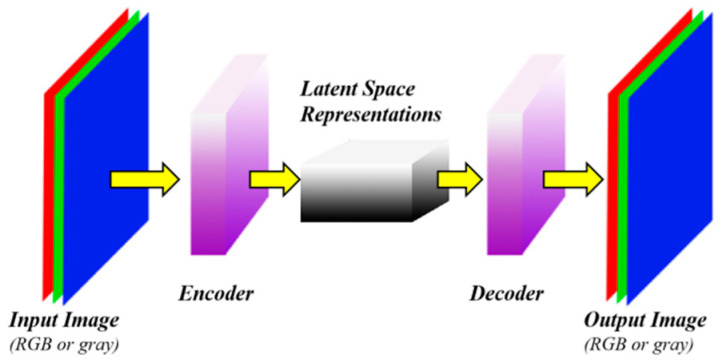
The structures of standard CDAE.

Convolutional autoencoder (CAE) combines the autoencoder and convolutional neural networks [42]. CDAE network is developed by DAE and CAE, and it belongs to an unsupervised learning method. CDAE accepts damaged input data for model training. It combines the convolution and pooling layers of the CNN, and adds an upsampling layer, which enables it to implement encoding and decoding operations. While achieving feature extraction, CDAE retains two-dimensional structure information of the image. The biggest advantage of CDAE lies in the weight sharing of the convolutional network structure and the local perception characteristics of the image. Weight sharing can effectively reduce the number of parameters that need to be trained in the network, and local perception feature can reflect the structure information of the image well. The typical CDAE network structure is shown in Figure 4.

Let *x* be the original vibration signal, α be the original image transformed by the signal, and $\hat{\alpha }$ be the converted image after the original signal adding noise, then:

(7) α=CWT(x(t)),

(8) $$\hat{\alpha }=CWT\left(x\left(t\right)+n\left(t\right)\right),$$

where n(t) is a noise signal conforming to a certain distribution.

Then for any *j*-th feature map, it can be defined by Equation (9):

(9) $$h^{j}=F_{\sigma }\left(\hat{\alpha }\right)=\varphi \left(\hat{\alpha }*W^{j}+b^{j}\right),$$

where $\sigma =\left\{W^{j},b^{j}\right\}$, ∗ represents the convolution operation, φ(⋅) represents the activation function, $W^{j}$ and $b^{j}$. are the weight matrix and deviation vector respectively, and the weight and deviation parameters are shared on the entire map.

The encoded image is reconstructed by the decoder, as shown in Equation (10):

(10) $$y=F_{\sigma^{\prime }}^{\prime }\left(h^{j}\right)=\varphi^{\prime }\left(\sum_{j\in N}h^{j}*{W\prime }^{j}+b^{\prime }\right).\;,$$

where $\sigma^{\prime }=\left\{W^{\prime j},b^{\prime j}\right\}$. , * represents convolution operation, $\varphi^{\prime }\left(\cdot \right)$. represents activation function, $W^{\prime j}$ and $b^{\prime j}$ are weight matrix and deviation vector, respectively *N* represents a set of potential feature maps.

Commonly used activation functions are ReLU and sigmoid functions, which are defined as:

(11) $$\mathrm{ReLU}\left(x\right)=\{\begin{array}{c} x,\;\;x>0\\ 0,\;\;x\leq 0 \end{array},$$

(12) $$sigmoid\left(x\right)=\frac{1}{1+e^{-x}},$$

The parameter optimization of σ and $\sigma^{\prime }$ is carried out by the back propagation algorithm. In order to effectively suppress the noise, the CDAE needs to be optimized to minimize the objective function, expressed as:

(13) $$J\left(\sigma ,\sigma^{\prime }\right)=\underset{\sigma ,\sigma^{\prime }}{argmin}\frac{1}{M}\sum_{i=1}^{M}L\left(y_{i},\alpha_{i}\right),$$

where *M* is the sample size and *L* is the loss function. Generally, the mean square error (MSE) loss function is used, then the objective function can be written as:

(14) $$J\left(\sigma ,\sigma^{\prime }\right)=\underset{\sigma ,\sigma^{\prime }}{argmin}\frac{1}{M}\sum_{i=1}^{M}\left(\frac{1}{2}\|y_{i}-\alpha_{i}\|{}^{2}\right),$$

Through the unsupervised training of the CDAE network, the noisy image can be restored to the original image after decoding, so it can improve the model’s ability to express denoising features.

## 3. The Proposed Method

In engineering practice, the operation of rolling bearings often has a lot of environmental noise, especially in high-speed and complex working conditions. In addition, the problems of training difficulty and performance degradation of deep CNN indicate that deep network is difficult to give full play to its unique and powerful learning ability. Based on the above two points, this paper proposes a new method for rolling bearing fault diagnosis based on residual dilated pyramid network and full convolutional denoising autoencoder (RDPN-FCDAE), which is dedicated to optimizing the training process of the network and extracting fault features covered by strong noise. This method will be described in detail in this section.

### 3.1. Transformation of Noise Signal to Gray Image Based on CWT

In the actual industrial environment, noise is a major issue that affects the performance of fault diagnosis. In order to verify the generalization ability of the model and better reflect the actual condition of the rolling bearing operation, additive white Gaussian noise (AGWN) with a specified signal-to-noise ratio (SNR) can be added to the signal to obtain a noisy signal, signal-to-noise ratio is defined as:

(15) $$\mathrm{SNR}=10{log}_{10}\left(\frac{P_{signal}}{P_{noise}}\right),$$

where $P_{signal}$. is the power of the signal and $P_{noise}$ is the power of the noise.

Wavelet analysis has a wide range of applications in mechanical fault diagnosis [43]. This paper uses CWT to convert the noise-containing signal into a time-frequency spectrum for analysis. The converted image contains enough fault information, which is helpful for fault classification.

There has been a lot of research on the effectiveness of mother wavelet functions and their ability to match various signals [44]. Among them, the Morlet wavelet has been proved by related research to be similar to the transient pulse component of bearing fault, which is superior to other wavelets in the analysis of non-stationary vibration signal of rolling bearings [45]. In this paper, a complex Morlet wavelet with a bandwidth of 3 Hz and a center frequency of 3 Hz is used as the mother wavelet function for rolling bearing fault diagnosis. The time domain formula is defined as follows:

(16) $$\psi \left(t\right)=\frac{1}{\sqrt{\pi f_{b}}}exp\left(-\frac{t^{2}}{f_{b}}+j2\pi f_{c}t\right),$$

In the formula, $f_{b}$ is the mother wavelet bandwidth, the value $f_{b}=3\;\mathrm{Hz}$; $f_{c}$ is center frequency, the value $f_{c}=3\;\mathrm{Hz}$.

The CWT using complex Morlet as the mother wavelet function can fully capture the signal characteristics and obtain good resolution in the time-frequency domain. The transformed wavelet time-frequency map is represented by a gray image, and the image is used as the input of the network model.

### 3.2. Dilated Pyramid Network

In order to obtain as large a receptive field as possible to extract more feature information to improve the diagnostic performance of the model, we introduce dilated convolution module in the network. However, from the mechanism of dilated convolution, it can be seen that when *r* > 1, its convolution kernel is not continuous, and the input information sampling is sparse, so it will produce a “grid effect”. For fault feature extraction of large targets, good results will be obtained, but local weak feature information will be lost.

**Figure 5:**
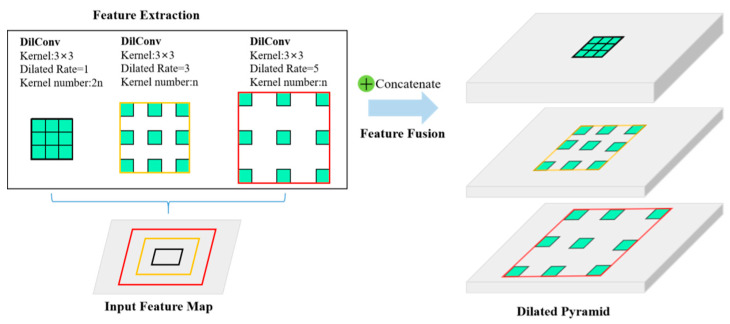
Dilated Pyramid Network (DPN). DPN obtains the multi-scale feature pyramid by exploiting multiple parallel filters with different dilated rates. The effective Receptive Field are shown in different colors.

Szegedy et al. proposed an inception module for image classification, which fully utilized the multi-scale information of the image [46]. GoogLeNet built based on the Inception module achieved the first place in the 2014 ImageNet competition. Inspired by this, we propose a multi-scale module stacking based on dilated convolution to form a feature pyramid structure, so that the network can learn not only the information of large targets, but also the local weak feature information, and the structure is shown in Figure 5. The pyramid structure uses convolution with kernel size of 3 × 3, dilated rate of *r* = 1, *r* = 3, and *r* = 5 for the input feature map. In addition, considering the balance of the feature information of the small target and the large target of the rolling bearing time-frequency map actually converted by CWT, the ratio of the number of convolution kernels of the three dilated rates is set as:

(17) n(r=1):n(r=3):n(r=5)=2:1:1,

After performing convolution operations, we then cascade feature maps of all dilated rates to form a pyramid structure to fuse feature information of different scales, thus forming a dilated pyramid network (DPN).

### 3.3. The Structure of RDPN-FCDAE

**Figure 6:**
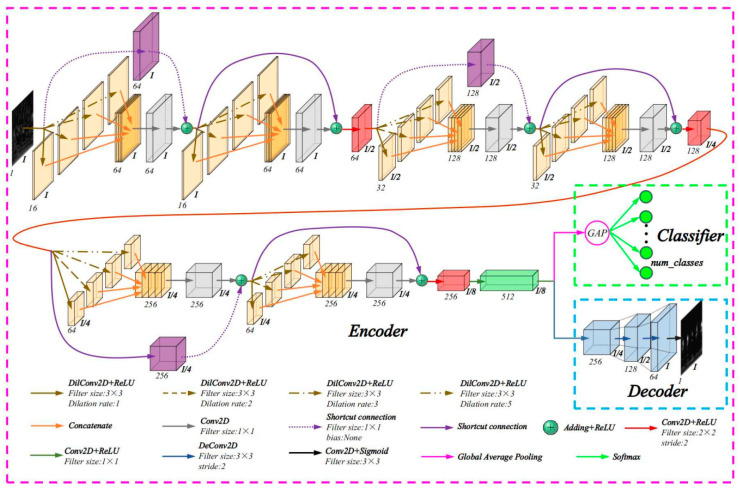
Architecture of the proposed RDPN-FCDAE model.

The structure and parameter settings of the RDPN-FCDAE model are shown in Figure 6. The model includes three parts: coding network, decoding network and classification network.

In the encoder network, the DPN module was first introduced to obtain feature information of multiple scales. After the DPN module, an additional convolution layer with kernel size of 1 × 1 is added. It has no activation function, and the purpose is to eliminate the feature fusion stacking effect. In order to optimize the network training, residual learning is introduced in this part to form a residual dilated pyramid network (RDPN) module. Of course, if the input and output dimensions do not match, the residual learning is done by a convolution operation with a kernel size of 1 × 1, which has no bias term and activation function. The ReLU activation function is added after each residual block. Finally, the calculation formula of each RDPN module is as:

(18) $$F_{1}=\mathrm{ReLU}\left(X*DilConv1\right),$$

(19) $$F_{2}=\mathrm{ReLU}\left(X*DilConv2\right),$$

(20) $$F_{3}=\mathrm{ReLU}\left(X*DilConv3\right),$$

(21) $$Y=\{\begin{array}{c} \mathrm{ReLU}\left(\left\{F_{1},F_{2},F_{3}\right\}*Conv1+X\right)\;\;\;\;\;if\;dimensions\;match\;\\ \mathrm{ReLU}\left(\left\{F_{1},F_{2},F_{3}\right\}*Conv1+X*Conv2\right)\;\;if\;dimensions\;not\;match \end{array},$$

where *X* is the input; *Y* is the output; * is the convolution operation; *DilConv* represents 3 different dilated rate convolution kernels; *Conv1* is a 1 × 1 convolution kernel that eliminates the fusion stacking effect, *Conv2* is a shortcut connection used to match the dimensions with 1 × 1 convolution kernel, *{·}* represents pyramid structure cascade algorithm.

**Figure 7:**
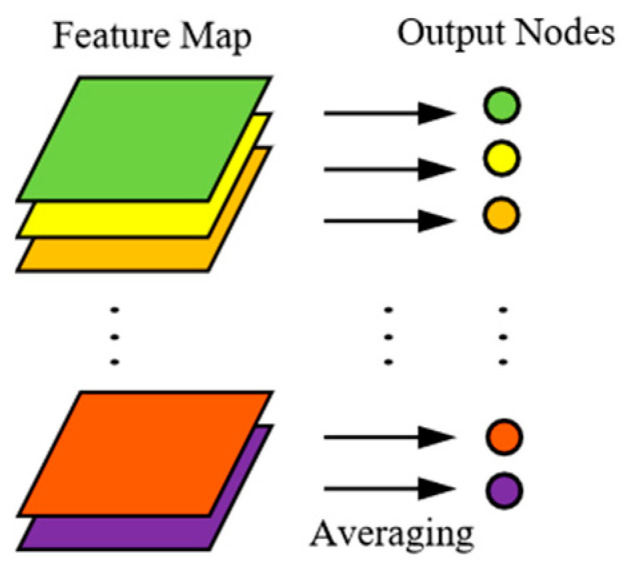
Architecture of GAP layer.

In order to reduce the loss of weak feature information, a cross-step convolution with kernel size of 2 × 2 and stride of 2 is introduced after every two RDPN modules instead of the largest pooling layer for dimension reduction. After six RDPN modules, 512 convolutional layers with kernel size of 1 × 1 were used to replace the fully connected layer. The decoding network first consists of three consecutive deconvolutional layers, followed by an output layer with a convolution kernel of size 3 × 3, and the activation function is Sigmoid. For the classification network, add a global average pool (GAP) at the top of the coding network. Figure 7 shows the architecture of the GAP layer.

The GAP layer can perform the fault classification task by taking the average value of each feature map and inputting it to the Softmax classification layer. The advantage of using GAP layer instead of fully connected layer is that it can reduce a large number of parameters, prevent overfitting, and the input size can be arbitrary, which is convenient for practical use. The Softmax function is represented by Equation (22):

(22) $$Q^{j}\left(z\right)=\frac{e^{z^{j}}}{\sum_{k=1}^{K}e^{z^{j}}},$$

Among them, *z* represents the *j*-th input feature of the Softmax activation function, and *k* is the number of fault categories.

### 3.4. Training

The model is a two-stage training model. In the first stage, which is the pre-training part, the model is composed of a coding network and decoding network. The input is a gray-scale time-frequency image obtained by CWT of the original vibration signal. If the input image is converted from a noise signal, then calculate the MSE loss of the clean image and the reconstructed image after the training sample passes the model. This process is unsupervised. Through the training of the encoding-decoding network, the model can automatically extract representative robust features from the input image.

In the second stage, the characteristics of the original data have been fully expressed due to the pre-training of parameters such as weight and bias in the first stage. In order to diagnose the rolling bearing faults, it is necessary to adopt fine-tuning strategy of parameters. At this point, the decoding network of the model is removed, and the classification network is added to the top layer of the coding network to form a residual dilated pyramid full convolution network (RDPFCN). The classification loss function uses cross entropy function to evaluate the error between the estimated probability distribution output by Softmax and the target class probability distribution. Let P(z) be the target distribution, and Q(z) be the estimated distribution, then the cross entropy between P(z) and Q(z) can be expressed by Equation (23):

(23) $$L=E\left(P\left(z\right),Q\left(z\right)\right)=-\sum_{j=1}^{K}P_{j}\left(z\right)logQ_{j}\left(z\right),$$

The fine-tuning process is a supervised learning process, which means to set the classification labels on the training data. After fine-tuning, save the model and input the test sample into the saved model to realize the fault diagnosis of rolling bearings.

The model training uses the gradient descent method and the mini-batch training method. In the calculation process, the error back propagation is used to calculate the gradient of the error function to all weights and offset values. The parameters and activation function settings of each layer of the model have been described in Figure 6. There are many algorithms for hyperparameter optimization [47]. In this paper, the adaptive moment estimation (Adam) algorithm with better comprehensive performance is used for optimization. In addition, in order to improve the training speed and shorten the training time, this paper uses graphics processing unit (GPU) calculation.

### 3.5. Implementation of Fault Diagnosis Algorithm

This paper presents a rolling bearing fault diagnosis algorithm based on RDPN-FCDAE. The general steps of the Algorithm 1 are shown below.
**Algorithm 1. General Steps of RDPN FCDAE Model.****Input:** Bearing datasets consisting of rolling bearing vibration signal samples**Output:** Diagnostic results, including classification accuracy, F-metrics, confusion matrix, feature segmentation, etc.**Step 1: data collection**1.1. Collect the vibration signal of rolling bearing through sensors, data acquisition system and host computer system.**Step 2: data preprocessing**2.1. Sort the collected original rolling bearing vibration signal datasets to obtain sample sets for each category, and add an additive white Gaussian noise with appropriate signal-to-noise ratio to each sample set signal to be closer to the actual working conditions.2.2. Use CWT to convert the signal into a time-frequency image and express it in grayscale.2.3. Normalize the obtained image, divide it into training set, verification set and test set according to a certain proportion, and do a good job of classification labeling.**Step 3: encoding-decoding training**3.1. Build the RDPN-FCDAE network structure, and randomly initialize the network weights and offsets in the form of Gaussian distribution.3.2. Encoding and decoding training of the proposed RDPN-FCDAE model on the training datasets in an unsupervised manner.**Step 4: Fine-tune the parameters**4.1. Remove the decoding network of RDPN-FCDAE model.4.2. Add GAP layer and Softmax classification layer on top of the coding network to form RDPFCN, and fine-tune the parameters through back propagation. This process is labeled.**Step 5: Verify algorithm performance**5.1. Verify the effectiveness of the algorithm on the test datasets and evaluate the diagnosis results.

## 4. Experimental and Results Analysis

### 4.1. Experimental Setup and Data Acquisition

**Figure 8:**
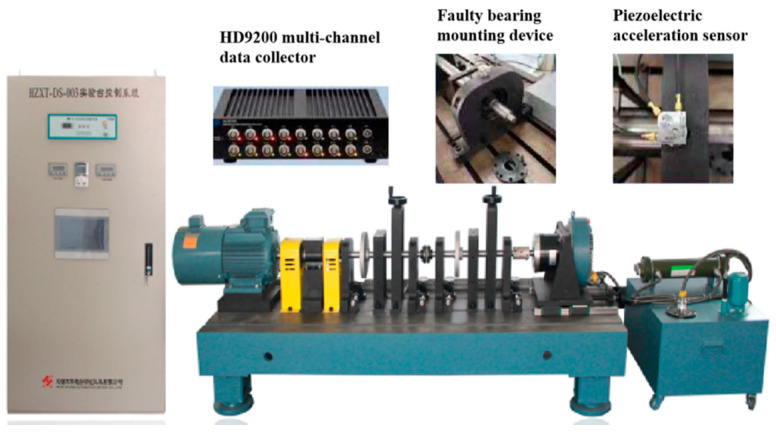
Double-span and double-rotor comprehensive fault simulation test rig.

Rolling bearing fault diagnosis experiment was carried out carried out on the experimental device as shown in Figure 8. It is a double-span and double-rotor comprehensive fault simulation test rig. The test rig is composed of T-slot pedestal, variable frequency motor and motor drive, single-span sliding bearing rotor system, single-span rolling bearing rotor system, coupling, sensor system and mounting bracket, etc. As shown in Figure 8, experimental bearing was mounted on bearing seat, and vibration signals were collected by different acceleration sensors mounted on bearing seat. The HD-YD-232 three-axis ICP piezoelectric sensor was selected as the acceleration sensor. This kind of sensor is widely used in vibration measurement of various equipment, and has the advantages of wide frequency response and small size. Finally, signal was transmitted to the host computer system through the HD9200 multi-channel data collector. The bearing type is NSK-6308 deep groove ball bearing, and the sampling frequency of the vibration signal is 20 KHz.

In order to verify the effectiveness of the proposed RDPN-FCDAE model, the experiment used electrical discharge machining (EDM) to implant artificial faults in the bearing inner ring raceway, outer ring raceway, rolling elements and cage, fully simulating multiple types of failure modes and mixed failure mode conditions. 

**Figure 9:**
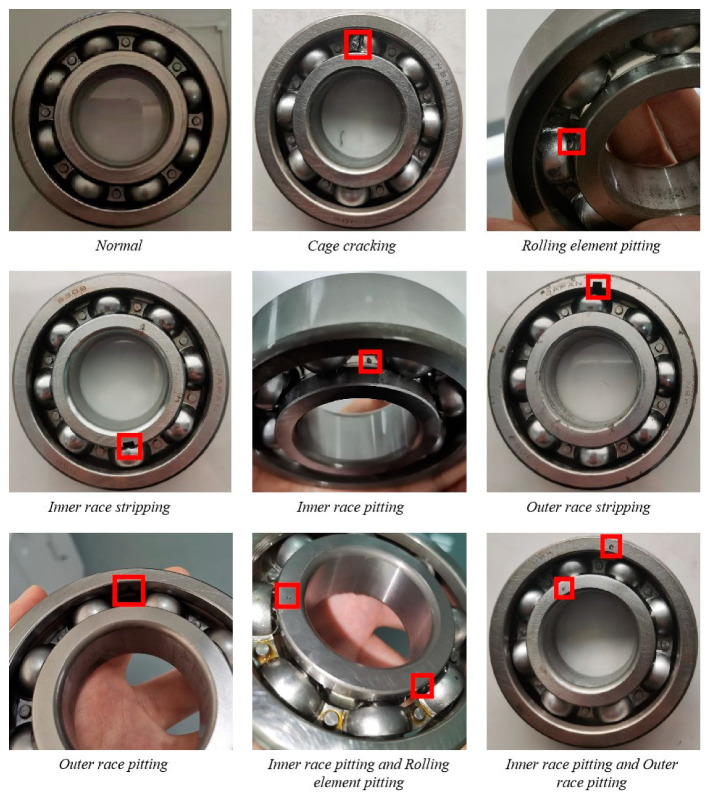
Schematic diagram of rolling bearings in nine health modes. The internal fault position of the rolling bearing corresponds to the marked position.

**Figure 10:**
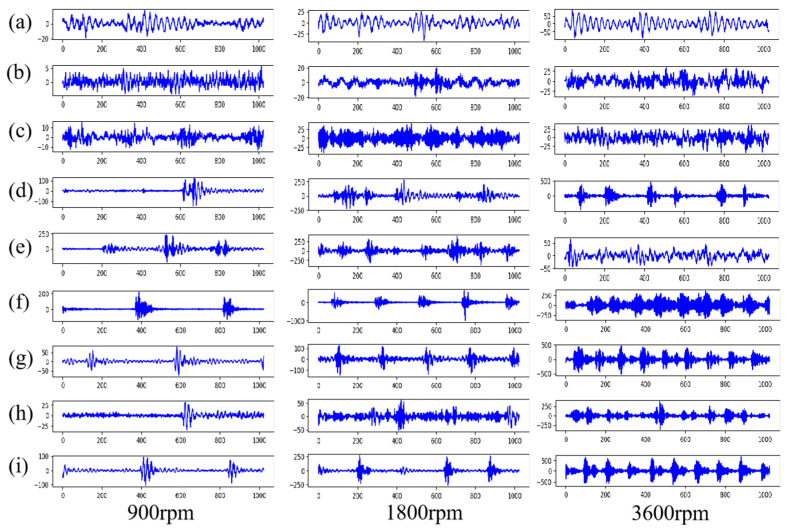
Vibration signals under the three bearing operating conditions. (**a**) Normal condition. (**b**) Cage cracking. (**c**) Rolling element Pitting. (**d**) Inner race stripping with a size of 4 mm × 0.3 mm. (**e**) Inner race pitting. (**f**) Outer race stripping with a size of 4 mm × 0.3 mm. (**g**) Outer race pitting. (**h**) Inner race and Rolling element pitting. (**i**) Inner race and Outer race pitting.

As shown in Figure 9, a total of nine kinds of bearing health conditions are simulated, including a normal mode, six single-type faults, and two mixed fault modes. The detailed information on various faults of rolling bearings is shown in Table 1. In order to simulate the working conditions of rolling bearings at different speeds as much as possible, the experiment designed three speed acquisition schemes of 900, 1800 and 3600 rpm for each health state. The original signals of each health mode collected are shown in Figure 10. 204,804 data points were collected in each state mode, and a total of 5,529,708 data points were collected. For each state mode, it is divided into training set, verification set and test set according to the ratio of 7: 2: 1, with 1024 data points as a vibration signal sample. Then each sample is converted into a time-frequency grayscale image by CWT. Each rotation speed is 27,000 image samples, including 21,000 samples in the training set, 6000 samples in the verification set, and 3000 samples in the test set. A total of 81,000 image samples at three speeds.

**Figure 11:**
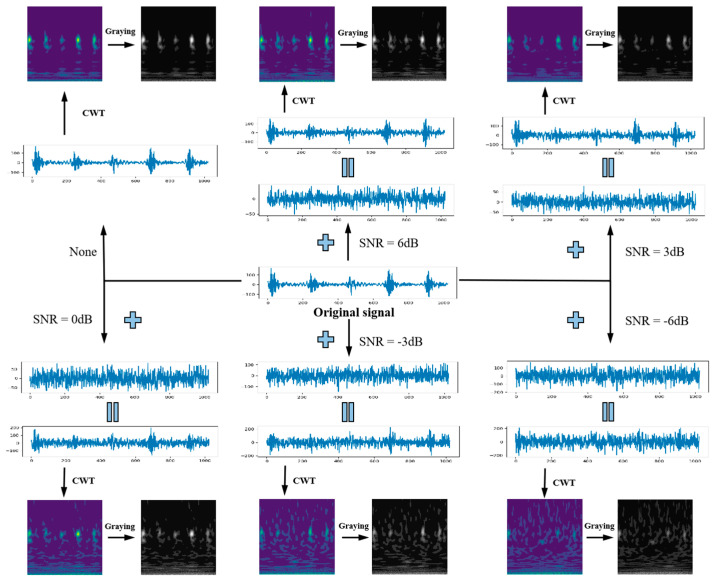
Schematic diagram of noise addition and CWT of original vibration signal of outer race pitting under 1800 rpm.

Due to the influence of the experimental environment and the device, the collected vibration signal inevitably contains a certain amount of noise. In order to better simulate the actual working conditions of rolling bearings, noises with SNR of −6 dB, −3 dB, 0 dB, 3 dB and 6 dB were added to each original signal sample, and then converted into time-frequency grayscale images by CWT. Taking 1800 rpm, the outer ring pitting vibration signal as an example, its noise adding and CWT conversion schematic diagram is shown in Figure 11.

A total of 486,000 image samples were obtained from the three rotation speeds, including 81,000 original signal image samples and 405,000 noise signal image samples. Table 2 is a summary table of datasets segmentation. The noise signal image is used as the input of the RDPN-FCDAE model, and the original signal image is used as the target output of the model.

The entire model uses Python3.7 as the programming language, Tensorflow1.14.0 as the deep learning algorithm programming framework, and the model training and testing on the NVIDIA GeForce GTX-1050Ti GPU, Lenovo, China. The PC operating system is ubuntu18.04.

### 4.2. Fault Diagnosis Results and Analysis

**Figure 12:**
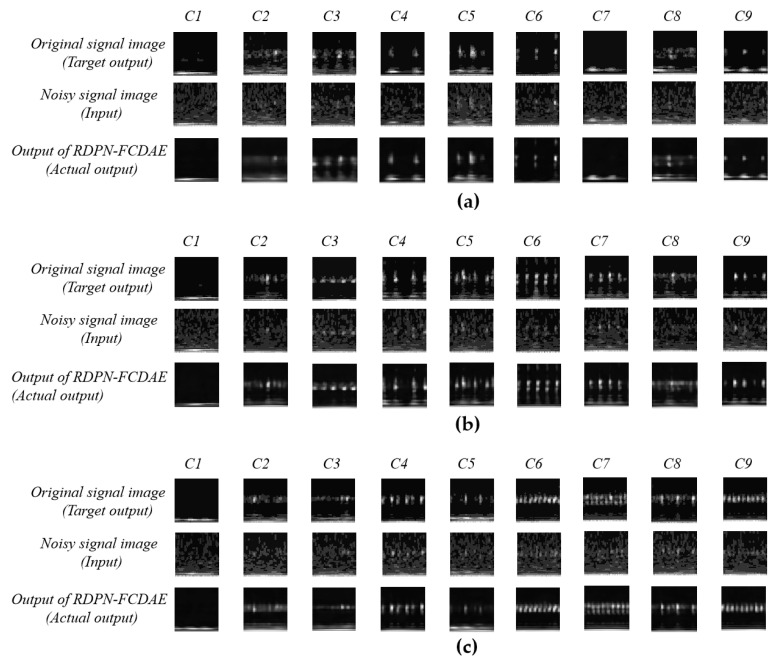
SNR = -6dB working condition, Under the condition of SNR = -6dB, the target output, input and actual output results of the RDPN-FCDAE model. (**a**) 900 rpm speed. (**b**) 1800 rpm speed. (**c**) 3600 rpm speed.

The experiment was conducted for three speed conditions. Firstly, the RDPN-FCDAE model was pre-trained. Figure 12 shows the comparison of model input, target output and real output after training 30 epochs at three speeds. Obviously, it can be seen from the figure that after the model training, the noise signal image is repaired, the noise is basically eliminated, and it has a very high similarity with the target output. This is due to the CDAE’s MSE loss function during the training process, which makes the noise image approximate to the original clean image. Therefore, the features extracted by the model can well express the fault denoising feature of rolling bearing.

Followed by fine-tuning the RDPFCN model and classification evaluation, since a single indicator cannot measure the classification performance more comprehensively, therefore, in order to comprehensively judge the quality of the model, this paper uses two indicators of accuracy and F1-measure to evaluate the classification performance. The mathematical definition is as Equations (24) and (25):

(24) $$Acc=\frac{TP+TN}{TP+TN+FP+FN}$$

(25) $$F1=\frac{2\times P\times R}{P+R},\;P=\frac{TP}{TP+FP},\;R=\frac{TP}{TP+FN})\;$$

Among them, *TP* is true positive, *FP* is false positive, *TN* is true negative, and *FN* is false negative. *Acc* is the accuracy rate, indicating the proportion of all samples that are correctly predicted; *P* is the accuracy, indicating the proportion of positive samples that are actually positive samples; *R* is the recall rate, which represents the proportion of positive samples that are correctly predicted as positive samples; *F1* represents the *F1*-measure, which is the harmonic average of accuracy and recall rate. 

**Figure 13:**
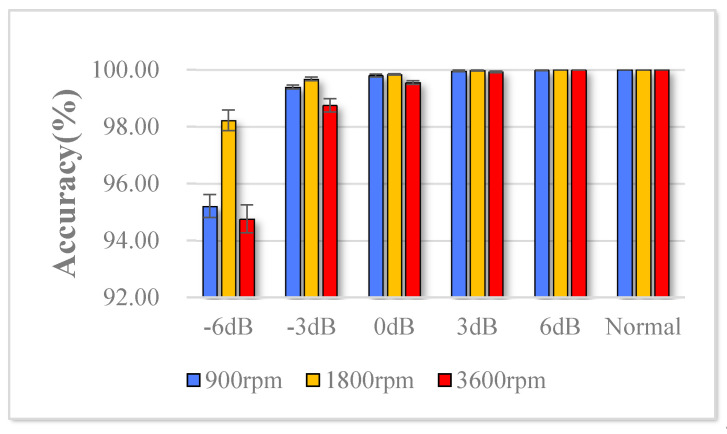
Diagnosis results of RDPN-FCDAE trained with different speed and SNR value.

The datasets of three rotation speeds and six noise-addition modes are classified, and 10 experiments are performed for each mode. The average classification accuracy and standard deviation results of the test set are shown in the Table 3, and the diagnosis results are shown in Figure 13. Experiments show that under the strong noise of SNR reaching –6 dB, the *Acc* is 95.21% at 900 rpm; 98.22% at 1800 rpm; 94.76% at 3600 rpm; Under SNR of –3, 0, 3 and 6 dB and normal conditions, the *Acc* at various speeds is greater than 98% or even 100%, which shows that the model can accurately predict rolling bearing failures in various changing environments. 

**Figure 14:**
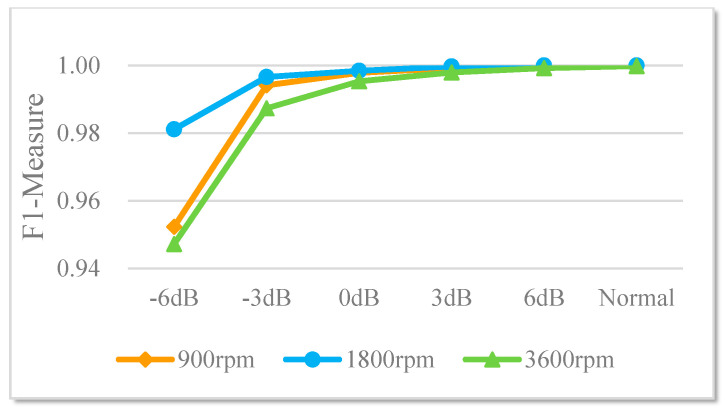
The trend of the F1-metric value with the change of the noise-adding mode.

The trend of the *F1*-measure value with the change of the noise-adding mode is shown in Figure 14. Its value is greater than 0.94 in various noise modes, and the closer the value is to 1, the stronger the fault diagnosis capability of the classifier for different modes of modes is.

**Figure 15:**
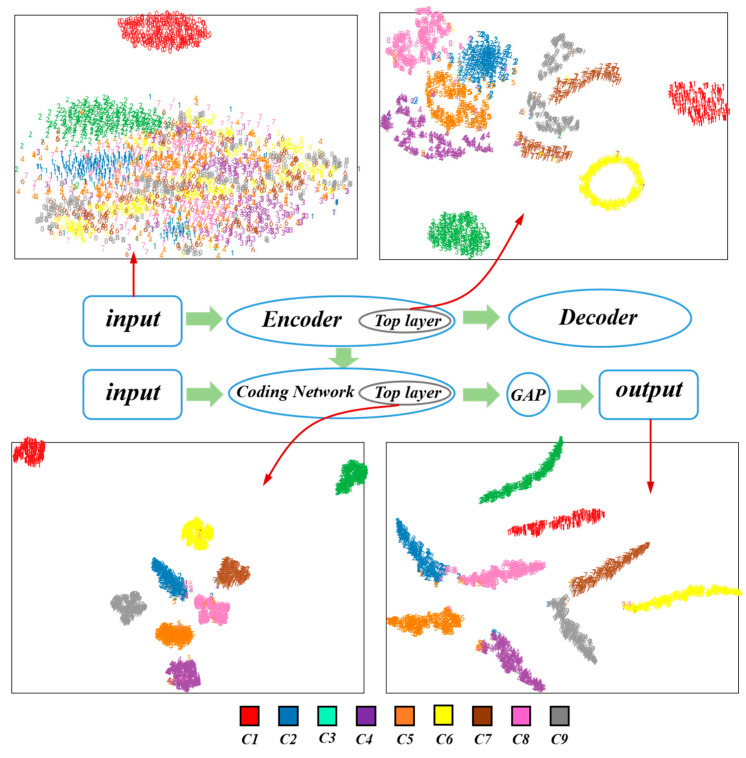
Visualization of different layer of the proposed RDPN-FCDAE.

The t-SNE technique [48] is an effective nonlinear dimensionality reduction algorithm that can be used to embed high-dimensional data in low-dimensional space for representation. In order to further describe the feature distribution of each layer of the RDPN-FCDAE model, taking 1800 rpm and SNR = −6 dB mode as an example, the t-SNE method is used for its input, the top layer of the pre-trained coding network, the top layer of the fine-tuned coding network, and the output layer feature distribution is visualized in two dimensions, as shown in Figure 15. It can be seen that the input features are disordered. After the model pre-training, the features have preliminary distribution rules. After fine-tuning, all kinds of features have been basically separated at the top layer of the coding network, and different types of faults have been clearly separated. Finally, the output layer features are further separated and distributed in a certain manifold.

### 4.3. The Necessity of Dilated Convolution and FCDAE

In order to verify the effect of the designed network tricks, this section designs an ablation experiment to research the network performance. At 1800 rpm and SNR = −6 dB, The diagnosis results of four schemes are compared: no use of FCDAE and dilated convolution, no use of FCDAE, no use of dilated convolution, and RDPN-FCDAE model, each scheme is tested 10 times. During training, the batch size is set to 128, the learning rate is 0.001, the network parameters are updated using the back propagation algorithm, and iterates 3000 times for classification. If the FCDAE scheme is used, pre-training 30 epochs with a batch size of 20.

**Figure 16:**
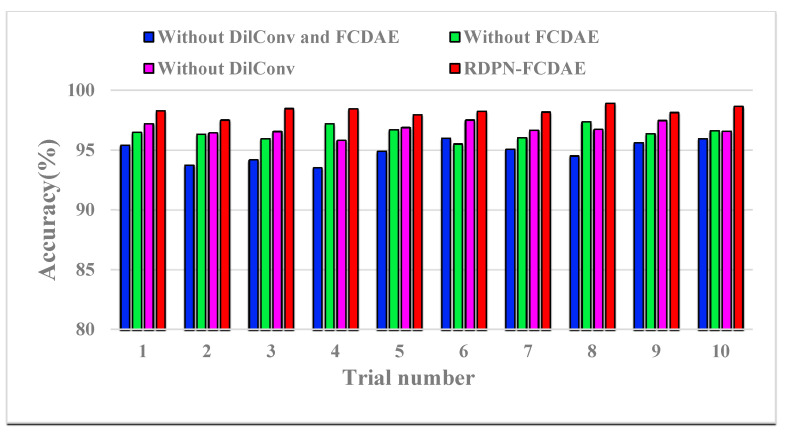
Ablation experiment results for ten trials (Speed = 1800 rpm, SNR = −6 dB).

The diagnosis results of 10 experiments are shown in the Figure 16, and the average accuracy and standard deviation are shown in the Table 4. 

It can be seen that when FCDAE and dilated convolution are not used, the average accuracy of 10 experiments is 94.8963%, the standard deviation is 0.8863. After using dilated convolution, the accuracy rate is 96.4630%, and the standard deviation is 0.5612, the diagnosis effect is significantly better. If not using dilated convolution, but using FCDAE for pre-training, the accuracy rate is 96.7926%, the standard deviation is 0.5148, and the diagnosis effect has been improved. When FCDAE and dilated convolution are used at the same time, the RDPN-FCDAE is constituted with an accuracy rate of 98.2889%, and the standard deviation is reduced to 0.3860. 

**Figure 17:**
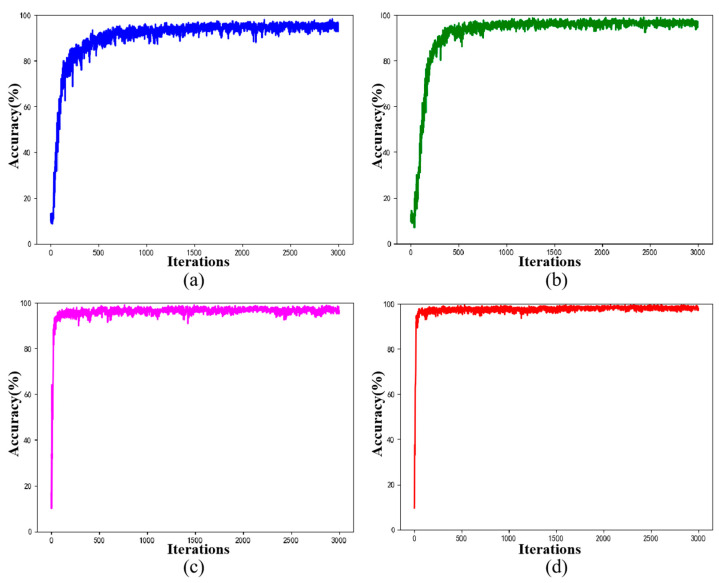
Accuracy curve of the verification set for 3000 iterations (Speed = 1800 rpm, SNR = −6 dB). (**a**) Without DilConv and FCDAE. (**b**) Without FCDAE. (**c**) Without DilConv. (**d**) RDPN-FCDAE.

Figure 17 shows the accuracy curve of the verification set for 3000 iterations of the four schemes in an experiment. It can be seen that the use of dilated convolution and FCDAE’s pre-training can significantly improve the diagnostic accuracy and reduce training fluctuation, when both are used at the same time, the diagnostic accuracy and training stability are further improved.

### 4.4. Comparison with Other Methods

**Figure 18:**
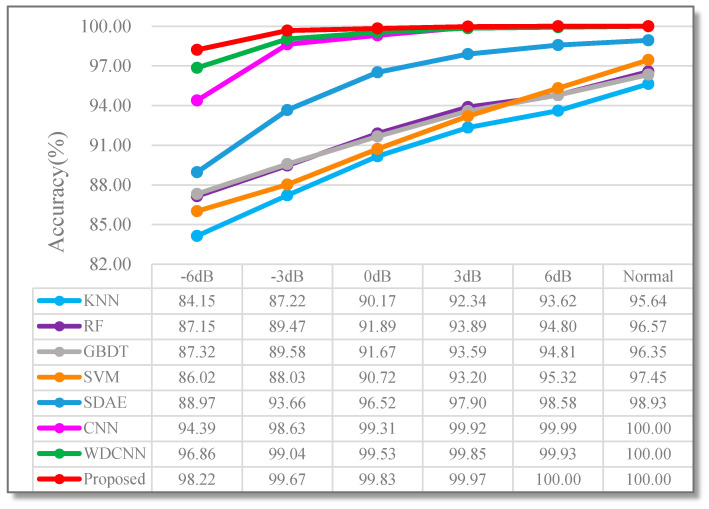
Comparison of performance testing of different methods under different SNR values (Speed = 1800 rpm).

In order to verify the superiority of the model, on the basis of traditional machine learning and deep learning, a total of 8 different classification algorithm comparison experiments were designed, which are KNN, RF, GBDT, SVM, stack denoising autoencoder (SDAE), CNN [20], WDCNN [35] and the proposed RDPN-FCDAE. The main parameters of these comparison algorithms are set as follows: (1) KNN: The input is 15 artificially extracted time-domain signal features. In each experiment, grid search is performed in the range of {3, 5, 9, 15} to find the optimal k value. (2) RF and GBDT: The input is 15 artificially extracted time-domain signal features, the number of weak classifiers is optimized from the range of {50, 100, 150, 200} using a grid search method and the decision tree is used as the weak classifier. (3) SVM: The input is 15 time-domain signal features manually extracted. A linear kernel function is selected, and the penalty factor C is searched from the range of {0.01, 0.1, 1, 10}. (4) SDAE: The input is the original time domain signal, the signal length is 1024, standardized pre-processing, the network structure is set to 1024-720-512-256-9, the training is divided into two stages of pre-training and fine-tuning. (5) CNN: The input is a normalized CWT time-frequency grayscale image, using the network structure and parameter settings of the paper [20]. (6) WDCNN: The input is the original time-domain signal, using the network structure and parameter settings of the paper [35]. (7) RDPN-FCDAE: The input is a normalized CWT time-frequency grayscale image, and its network structure and parameter settings are shown in Chapter 3. At a speed of 1800 rpm, different methods were used for 10 experiments in different noise modes and the average accuracy of the test set was taken as the final result. The comparison experiment results are shown in Figure 18.

Obviously, the accuracy of rolling bearing fault diagnosis based on traditional machine learning algorithm is significantly lower than that based on deep learning, which is mainly due to the shallow architecture of machine learning method, the diagnosis performance largely depends on artificial feature extraction, unable to deeply mine the complex features between fault category and signal data. Compared with the other three deep learning algorithm models, the method proposed in this paper has better diagnosis accuracy.

In order to further explore the diagnostic effects of the method proposed in this paper and the other three deep learning methods, taking the noise mode with a speed of 1800 rpm and an SNR of -6 dB as an example, the four methods are further analyzed.

**Figure 19:**
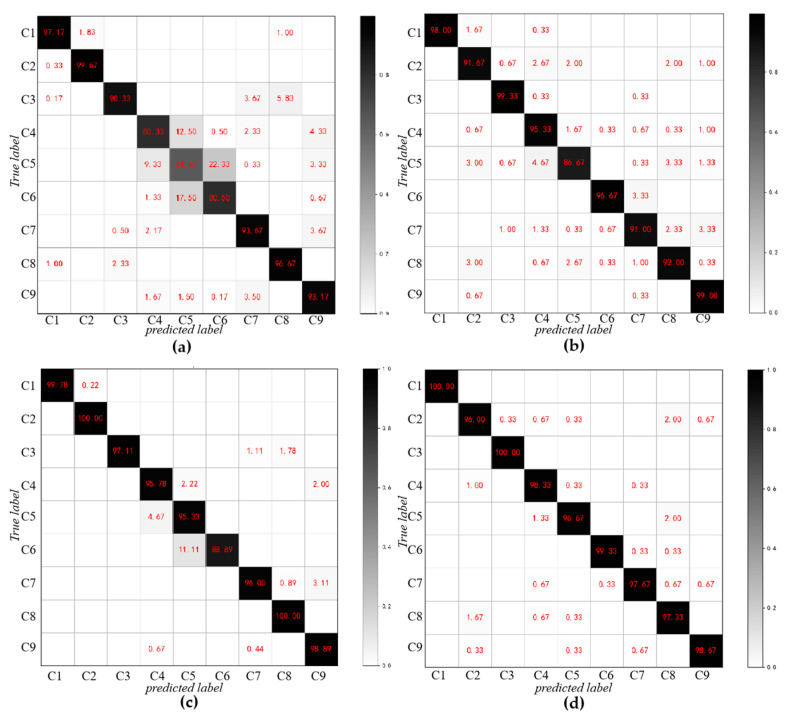
Comparison of multi-class confusion matrix for different deep learning methods (Speed = 1800 rpm, SNR = −6 dB). (**a**) SDAE. (**b**) CNN. (**c**) WDCNN. (**d**) RDPN-FCDAE (Proposed method).

**Figure 20:**
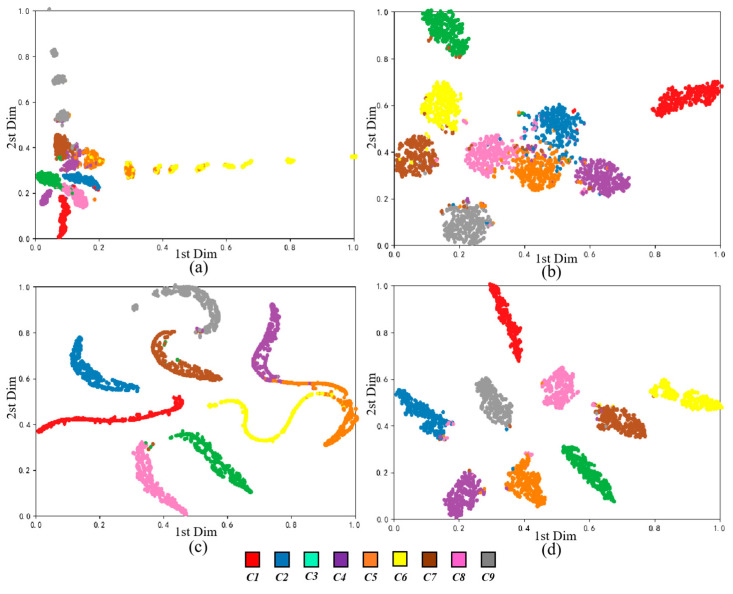
Comparison of feature segmentation performance of different deep learning methods (Speed = 1800 rpm, SNR = −6 dB). (**a**) SDAE. (**b**) CNN. (**c**) WDCNN. (**d**) RDPN-FCDAE (Proposed method).

Figure 19 lists the confusion matrix of these four algorithms on the test set. It can be seen that SDAE performs poorly on the classification of the fourth, fifth and sixth categories, especially the classification results of the fifth category samples are poor, and more than 30% are misclassified into other categories. The accuracy of various types of SDAE has mostly increased. CNN and WDCNN has effectively improved the classification effect, and the accuracy rate of the fifth classification has reached 86.67% and 95.33%, which is largely due to the advantages of convolution operations in image processing. The RDPN-FCDAE further improves the diagnosis effect, and the classification accuracy rate for all categories is above 95%, and the samples are basically correctly identified and classified. Figure 20 is the t-SNE dimensionality reduction visual analysis of the output layers of various algorithms on the test set, which reduces the high-dimensional data to the two-dimensional plane and normalizes it. The abscissa is one of the dimensions after dimensionality reduction to a two-dimensional space, and the ordinate is another dimension. Obviously compared to the other three methods, RDPN-FCDAE has the best feature segmentation performance.

## 5. Conclusions

In this paper, a rolling bearing fault diagnosis algorithm based on RDPN-FCDAE is proposed for rolling bearings working in a complex and variable environment with different noises and different speeds. According to the various characteristics of rolling bearing signals, the algorithm model flexibly designs the network structure. In order to obtain different levels of fault information, including obvious and weak fault features, a feature fusion pyramid structure based on multi-scale dilated convolution is designed. In order to improve the performance of the network, a residual connection is designed. In order to better improve the model’s deep plasticity and robustness to noise, a two-stage full convolutional denoising autoencoder training process is designed.

The diagnostic performance of the model was verified by the collected rolling bearing experimental data. The data collected under the three rotation speeds were analyzed by noise experiments and a visual analysis of the training results by t-SNE, which verified the noise robustness and speed adaptability of the model. Through ablation experiments, the rationality of the model structure design was verified. By comparing with other traditional machine learning methods and deep learning methods, the diagnostic superiority of this model is further proved. It is a very meaningful work to apply this model to the fault diagnosis of rolling bearings. In addition, because the model requires pre-training, although it plays a great role in noise suppression, it will also increase the calculation cost accordingly. In future research, the authors will further study a more lightweight fault diagnosis algorithm.

## Figures and Tables

**Table 1:** Detailed information on various faults of rolling bearings.

Location	Fault Description	Speed	Class
None	Normal	900 rpm, 1800 rpm, 3600 rpm	C1
Cage	Cracking		C2
Rolling element	Pitting		C3
Inner race	Stripping with a size of 4 mm × 0.3 mm		C4
Inner race	Pitting		C5
Outer race	Stripping with a size of 4 mm × 0.3 mm		C6
Outer race	Pitting		C7
Inner race and Rolling element	Pitting		C8
Inner race and Outer race	Pitting		C9

**Table 2:** Detailed information on various faults of rolling bearings.

Motor Speed (rpm)	SNR (dB)	Number of Classes	Number of Samples (Training Set: Validation Set: Test Set)	Total Samples
900	Normal、−6、−3、0、3、6	9	6 × 9 × (2100:600:300)	162,000
1800	Normal、−6、−3、0、3、6	9	6 × 9 × (2100:600:300)	162,000
3600	Normal、−6、−3、0、3、6	9	6 × 9 × (2100:600:300)	162,000

**Table 3:** The accuracy and Standard deviation of RDPN-FCDAE trained with different speed and SNR value.

Speed (rpm)			SNR (dB)			Normal
	−6	−3	0	3	6	
900	95.21 ± 0.3993	99.39 ± 0.0682	99.79 ± 0.0553	99.92 ± 0.0305	99.97 ± 0.0191	99.99 ± 0.0117
1800	98.22 ± 0.3603	99.67 ± 0.0616	99.83 ± 0.0250	99.97 ± 0.0209	100.00 ± 0.00	100.00 ± 0.00
3600	94.76 ± 0.4914	98.74 ± 0.2324	99.55 ± 0.0653	99.81 ± 0.0392	99.92 ± 0.0245	99.98 ± 0.0195

**Table 4:** Average accuracy and standard deviation of Ablation experiment for ten trials (Speed = 1800 rpm, SNR = −6 dB).

Method	Average Testing Accuracy (%)	Standard Deviation
Without DilConv and FCDAE	94.8963	0.8863
Without FCDAE	96.4630	0.5612
Without DilConv	96.7926	0.5148
RDPN-FCDAE	98.2889	0.3860
